# Supplementary material for: The Snowmelt Niche Differentiates Three Microbial Life Strategies That Influence Soil Nitrogen Availability During and After Winter
Source: Front Microbiol. 2020 May 15;11:871. doi: 10.3389/fmicb.2020.00871 (PMC7242569; doi:10.3389/fmicb.2020.00871)
Supplement: TABLE S1 — Daily soil freeze-thaw cycles measured in the Hillslope and Floodplain during winter and before and after snowmelt in 2016/17. We considered a freeze-thaw cycle to have occurred if the daily maximum soil temperature was > 0°C and the daily minimum soil temperature was < 0°C. [file Table_1.docx]

|  |  | Winter | Snowmelt | Spring |
| --- | --- | --- | --- | --- |
| Hillslope | FTCs (6 cm) | 11 | 0 | 0 |
|  | FTCs (17 cm) | 6 | 0 | 0 |
|  |  |  |  |  |
| Floodplain | FTCs (6 cm) | 3 | 0 | 0 |
|  | FTCs (17 cm) | 0 | 0 | 0 |

**Supplemental Table 1**. Daily soil freeze-thaw cycles measured in the Hillslope and Floodplain during winter and before and after snowmelt in 2016/17. We considered a freeze-thaw cycle to have occurred if the daily maximum soil temperature was > 0 °C and the daily minimum soil temperature was < 0 °C
