# Supplementary material for: The Snowmelt Niche Differentiates Three Microbial Life Strategies That Influence Soil Nitrogen Availability During and After Winter
Source: Front Microbiol. 2020 May 15;11:871. doi: 10.3389/fmicb.2020.00871 (PMC7242569; doi:10.3389/fmicb.2020.00871)
Supplement: Supplementary file 5 [file Data_Sheet_1.docx]

*Illumina Library Prep and PCR Conditions*

The hyper-variable V4 region of the 16S rRNA gene was amplified for identification of bacteria and archaea and the internal transcribed spacer region (ITS) was amplified for fungal identification. Forward and reverse PCR primers (bacteria and archaea 515F-806R; fungi ITS1F-ITS2R) were modified to include the forward and reverse Illumina Nextera adapters and the reverse PCR primer included a 12-bp Golay barcode. The primer constructs have been described in detail previously (Caporaso et al. 2012, Smith and Peay 2014). PCR reactions were performed in triplicate in 25 µL reactions with the following reagent concentrations, Takara Ex Taq (0.025 units µL-1), 1X Takara Ex Taq PCR buffer, Takara dNTPs mix (200 µM), Roche bovine serum albumin (0.56 mg mL-1), PCR primer (200 nM) and approximately 10 ng ul^-1^ DNA template. Bacterial amplification was completed with the following thermocycler settings, 95 °C for 3 min, 25 cycles of 95 °C for 45 s, 50 °C for 60 s, and 72°C for 90 s with a final extension of 10 min at 72 °C and for fungi 95 °C for 3 min, 30 cycles of 95 °C for 30 s, 51 °C for 30 s, and 72°C for 30 s with a final extension of 5 min at 72 °C. PCR products were purified using Sera-Mag (Thermo Scientific; Fremont, CA, USA) Solid-Phase Reversible Immobilization (SPRI) paramagnetic beads (DeAngelis et al. 1995, Fisher et al. 2011) . Purified PCR products were quantified using the Qubit hs-DS-DNA kit (Invitrogen; Carlsbad, CA) and pooled in equimolar concentrations and sequenced on a single lane for 300 bp paired-end Illumina v3 MiSeq sequencing completed at the Vincent J. Coates Genomics Sequencing Laboratory at UC Berkeley (Berkeley, CA).

**References**

Caporaso, J. G., C. L. Lauber, W. A. Walters, D. Berg-Lyons, J. Huntley, N. Fierer, S. M. Owens, J. Betley, L. Fraser, M. Bauer, N. Gormley, J. A. Gilbert, G. Smith, and R. Knight. 2012. Ultra-high-throughput microbial community analysis on the Illumina HiSeq and MiSeq platforms. Isme Journal **6**:1621-1624.

DeAngelis, M. M., D. G. Wang, and T. L. Hawkins. 1995. Solid-phase reversible immobilization for the isolation of PCR products. Nucleic Acids Res **23**:4742-4743.

Fisher, S., A. Barry, J. Abreu, B. Minie, J. Nolan, T. M. Delorey, G. Young, T. J. Fennell, A. Allen, L. Ambrogio, A. M. Berlin, B. Blumenstiel, K. Cibulskis, D. Friedrich, R. Johnson, F. Juhn, B. Reilly, R. Shammas, J. Stalker, S. M. Sykes, J. Thompson, J. Walsh, A. Zimmer, Z. Zwirko, S. Gabriel, R. Nicol, and C. Nusbaum. 2011. A scalable, fully automated process for construction of sequence-ready human exome targeted capture libraries. Genome Biol **12**:R1.

Smith, D. P., and K. G. Peay. 2014. Sequence Depth, Not PCR Replication, Improves Ecological Inference from Next Generation DNA Sequencing. PLoS ONE **9**:e90234.
